# The Splicing Variant TFIIIA-7ZF of Viroid-Modulated Transcription Factor IIIA Causes Physiological Irregularities in Transgenic Tobacco and Transient Somatic Depression of “Degradome” Characteristic for Developing Pollen

**DOI:** 10.3390/cells11050784

**Published:** 2022-02-23

**Authors:** Jaroslav Matoušek, Gerhard Steger

**Affiliations:** 1 Biology Centre of the Czech Academy of Sciences, Department of Molecular Genetics, Institute of Plant Molecular Biology, Branišovská 31, 37005 České Budějovice, Czech Republic; jmat@umbr.cas.cz; 2 Institutfür Pysikalische Biologie, Heinrich Heine University Düsseldorf, 40204 Düsseldorf, Germany

**Keywords:** *Nicotiana tabacum*, viroid, plant transformation, nucleolytic enzymes, plant aging, plant morphology changes, transcription factors

## Abstract

Viroids are small, non-coding, pathogenic RNAs with a significant ability of adaptation to several basic cellular processes in plants. TFIIIA-7ZF, a splicing variant of transcription factor IIIA, is involved in replication of nuclear-replicating viroids by DNA-dependent polymerase II. We overexpressed *Nb*TFIIIA-7ZF from *Nicotiana benthamiana* in tobacco (*Nicotiana tabacum*) where it caused morphological and physiological deviations like plant stunting, splitting of leaf petioles, pistils or apexes, irregular branching of shoots, formation of double-blade leaves, deformation of main stems, and modification of glandular trichomes. Plant aging and senescence was dramatically delayed in transgenic lines. Factors potentially involved in viroid degradation and elimination in pollen were transiently depressed in transgenic leaves. This depressed “degradome” in young plants involved *Nt*Tudor S-like nuclease, dicers, argonoute 5, and pollen extracellular nuclease I showing expression in tobacco anthers and leaves. Analysis of the “degradome” in tobacco leaves transformed with either of two hop viroids confirmed modifications of the “degradome” and TFIIIA expression. Thus, the regulatory network connected to TFIIIA-7ZF could be involved in plant pathogenesis as well as in viroid adaptation to avoid its degradation. These results support the hypothesis on a significant impact of limited TFIIIA-7ZF on viroid elimination in pollen.

## 1. Introduction

Transcription factor IIIA (TFIIIA) is a special member of the zinc finger family of nucleic acid-binding proteins with multiple functions. Its N-terminal polypeptide binds sequence-specifically DNA and RNA; its C-terminal peptide is involved in the transactivation process, possibly by interacting with other general factors [1]. TFIIIA is essential for the DNA-dependent RNA polymerase III-based transcription of 5S rRNA. It binds both 5S rDNA and 5S rRNA [2,3]. The TFIIIA gene contains a 5S rRNA-derived exon in all representative land plant species [4,5]. Skipping of this exon leads to the full TFIIIA protein with nine zinc fingers (TFIIIA-9ZF). Inclusion of this exon would lead to a protein with only the first two zinc fingers due to a premature stop codon (PTC), but this long mRNA is degraded as a target of nonsense mediated decay. In addition, a TFIIIA with only the last seven zinc fingers (TFIIIA-7ZF) is produced in dependence upon plant development [6,7]. Recent results suggest that viroids—small, circular, non-coding pathogenic RNAs with high adaptability to the metabolism of their plant hosts (for reviews see e. g., [8,9,10])—adopted TFIIIA-7ZF for their own propagation. Although TFIIIA and its shorter variant are modulated upon viroid infection and are obviously potent interactive members of the zinc finger family in plants, little is known about a possible involvement of TFIIIA-7ZF in physiological processes connected to viroid propagation, pathogenesis, or plant morphogenesis.

We already reported a possible involvement of the TFIIIA pathway in viroid elimination from pollen [11,12]. Specifically, we analyzed elimination of the two pollen- and seed non-transmissible hop viroids apple fruit crinkle viroid (AFCVd) and citrus bark cracking viroid (CBCVd). We concluded that there are at least two processes related to viroid elimination in infected maturing tobacco pollen; these are (i) viroid degradation and (ii) depressed viroid replication, possibly due to a lack of TFIIIA-7ZF [11]. We hypothesized that these simultaneous processes make viroid elimination from pollen efficient [11]. Our assumption about TFIIIA-7ZF was based on the previous finding that TFIIIA mRNA can be changed post-transcriptionally due to modified mRNA processing [4,5]. This processing regulation is influenced by viroid infection and, as a result, a shorter splicing variant TFIIIA-7ZF is generated for review see [7]. This modified variant is involved in PSTVd replication [13,14]. Specifically, pollen shows abundant translation due to active status of ribosomal regulation. Thus, a high level of ribosomal protein L5, the suppressor of TFIII-7ZF biogenesis, is present. Consequently, a relatively low level of TFIIIA-7ZF was assumed to accumulate in maturing pollen [11].

In our previous experiments authentic *Nb*TFIIIA-7ZF was cloned from *N. benthamiana* infected with potato spindle tuber viroid (PSTVd) strain AS1, one of the most lethal viroids [15]. Subsequently, *Nb*TFIIIA-7ZF co-expression with AFCVd was analyzed in a transient expression system [11]. More abundant levels of longer-than-unit-length viroid replication intermediates were indeed detected using Northern blot analyses, suggesting some positive impact of this modified factor onto AFCVd replication. In addition it was shown that co-expression of *Nb*TFIIIA-7ZF is able to influence the activity of other transcription factors (TF) and their complexes. This suggests additional function(s) of *Nb*TFIIIA-7ZF in viroid propagation and pathogenesis [11].

In the present study we used plant transformation of the viroid-symptomless model plant tobacco to study impact of ectopic overexpression of *Nb*TFIIIA-7ZF and found additional physiological features of viroid-modified TFIIIA, which could mediate viroid adaptations and pathogenesis.

## 2. Materials and Methods

### 2.1. Plant Cultivation Conditions, Plant Transformation and Sampling

*Nicotiana tabacum* (cv. Samsun; Institute of Experimental Botany, CAS, Czech Republic) plants were grown in big pots under greenhouse conditions at the Biology Centre, CAS České Budějovice, Czech Republic, in the seasons 2020 and 2021 to collect leaf samples, anthers, and pollen. In these conditions plants usually reached mass flowering after 90 days cultivation. Anthers and pollen were collected within the seasonal periods from July to August. Immature pollen was isolated and mature pollen was germinated as described previously [11], the isolation of pollen from fresh anthers in particular stages was accomplished [16] using length and morphology of flower buds of *N. tabacum* for developmental stage selection. In other plant cultivations performed to analyze tobacco leaves, plants were grown in smaller pots in climate boxes at a temperature of 25 ± 3 °C with supplementary illumination (90 μmol/m^2^/sPAR) to keep a 16h-day length. Under these conditions plants reached the stage of flowering usually after 70 days of cultivation.

*Agrobacterium tumefaciens* LBA4404-mediated transformation of *N. tabacum* with *Nb*TFIIIA-7ZF (Supplementary Figure S1) was performed using the vector pJM14 prepared previously [11]; see Figure S2 for a detailed scheme of the expression cassette. Vector pJM14 was modified on the basis of pBin-35S-mGFP5 [17,18], provided by Prof. David C. Baulcombe (Sainsbury Laboratory, John Innes Centre, Norwich, UK). Bright expression of mGFP5 integrated in this vector enabled us to perform clear pre-selection of plant transformants using GFP marker at early stages. Four independent plant lines strongly expressing GFP were pre-selected for further analyses and plant sampling. In some experiments we made pre-selection of seedlings derived from TFIIIA-7ZF transgenic lines after self-crossing. Mixed samples were prepared by pooling equal amounts of leaf cuttings from selected transgenic lines. Transformed/viroid-infected tobacco plants were prepared by *Agrobacterium* LBA4404, bearing infectious plant vectors designated (AFCVd)${}_{2}$ in pFAST and (CBCVd)${}_{2}$ in pFAST, containing dimeric (++) AFCVd and CBCVd cDNAs, respectively. See Figure S5 for more detailed schemes of the expression cassettes. The pFAST vector, provided by the laboratory of Prof. David Honys (Institute of Experimental Botany, ASCR, Prague, Czech Republic), was modified previously [11]. AFCVd was integrated into the unique *Kpn*I site (Figure S5a) and CBCVd into the unique *Spe*I site downstream pollen-specific promoter pLAT52 (Figure S5b). The transformation vectors enabled pre-selection using GFP marker. Transformed lines were checked for viroid infection using dot-blot hybridization (see below). Five transgenic lines were used for preparation of mixed samples for quantification of viroid and mRNA analyses.

Transformation of tobacco with TFIIIA-7ZF or with plant infectious vectors bearing either AFCVd or CBCVd was performed using the standard leaf disc method [19]. Regenerated transformed plants were maintained on medium containing 100mg/L kanamycin and 200mg/L timentin. Well-rooting lines on kanamycin were transferred from in vitro to the soil and subjected to further analyses.

### 2.2. Viroid Inoculation, Detection and Quantification

AFCVd (GenBank AC AB104533) or CBCVd (AC KM211547) were used for inoculation or transformation/infection of *N. tabacum* [20]. AFCVd inoculum was prepared as native viroid RNA isolated from infected host plant *N. benthamiana*, partly purified as 2M LiCl-soluble RNA fraction and inoculated using the biolistic method [11]. Detection of viroid in inoculated or transformed plants was performed using dot-blot hybridization [21] using crude RNA extracts from plant leaves in AMESS buffer (1M sodium acetate pH 6.0 containing 1M NaCl, 10mM MgCl${}_{2}$, 20% ethanol and 3% SDS) [22]. Full-length AFCVd or CBCVd ${}^{32}$P[dCTP]-labeled probes were used for viroid detection. Membranes from dot-blots were scanned using the Typhoon PhosphoImager (Amersham Biosciences, Sunnyvale, CA, USA). For viroid detection on Northern blots and for quantification, total RNA was isolated from 200mg of leaves (prepared as random mixture of cuttings from at least six young leaves or as mixed samples, see above) with Concert™ reagent (Plant RNA Purification Reagent, Invitrogen, Carlsbad, CA, USA) according to manufacture’s protocol followed by a DNA cleavage step.

For detection of AFCVd, Northern blot analyses were essentially performed as described previously [11]: RNA was electrophoresed on 1.5% formaldehyde-denaturating agarose gel, blotted onto Biodyne A transfer membrane (Pall, Hampshire, England) and hybridized to AFCVd cDNA probe.

For simultaneous RT-qPCR quantification of viroid (+) and (−) strands forming thermodynamically stable structures, we used singlestrand-specific RT-qPCR method described previously [20] as a duplex reaction in direct combination with amplification of 7SL RNA as control marker [11,12]. Due to the similar size, cellular levels, and structures of 7SL RNA and viroids, the same RT-qPCR conditions with *Tth* polymerase could be used, which made the method faster and more exact. In the first step, thermostable *Tth* polymerase was used for reverse transcription at 70 °C of viroid RNA (+) chains using primers AFCVdRTPL and CVdRTPL for AFCVd and CBCVd, respectively, or (−) chains with AFCVdRTMI and CVdRTMI primers (Table S1) for AVCVd and CBCVd, respectively. The individual primers in these first reactions were combined with anti-β primer (Table S1) to generate simultaneously the first strand of 7SL RNA marker. In the second step, real-time PCR amplification was performed with the cDNAs using PCR_FOR and PCR_REV primer combinations (Table S1) for AFCVd and CBCVd, respectively, and primer-α in combination with anti-β for 7SL RNA. The second duplex step to amplify viroid and 7SL RNA proceeded using “Q™ SYBR® Green Super-mix” (Bio-Rad, Hercules, CA, USA) [11] with initial denaturation at 94 °C for 4 min, 40 amplification cycles (94 °C/20 s, 61 °C/40 s, 72 °C/30 s).The quantification was performed on the CFX Connect™ Real-Time PCR Detection System (Bio-Rad) with Bio-Rad CFX Maestro qPCR software v1.1. Relative viroid levels were normalized with the “delta-delta method” [23] to the levels of 7SL RNA [11,12]. We calculated *p*-values for level of significance using the two-tailed *t*-test as in previous analyses [20].

### 2.3. Quantification of mRNA Levels of Genes Potentially Connected to Viroid Degradation and Markers of Senescence

For analysis of mRNA levels, 10 μg of purified and DNase-treated total RNA was reverse transcribed using oligo dT18 primer and Superscript III reverse transcriptase (Invitrogen, Carlsbad, CA, USA) at 50 °C for 60 min. Reactions were performed [20] with *Nt*TUDOR S-like nuclease (TSN), *Nt*argonaute 5 (AGO5) and DICER-like (DCL) primers selected in our previous work [11] as listed in Table S1. The following annealing temperatures were applied (across slashes): *Nt*AGO5/55.6 °C, *Nt*TSN/58 °C, *Nt*DCL homologues/54 °C. Note that the *Nt*DCL primers do not differentiate between DCL1–4. Conserved primers to detect variants of TFIIIA/56 °C were designed in this work for amplification of *Nt*TFIIIA-9ZF, *Nt*TFIIIA-7ZF, and *Nb*TFIIIA-7Z. Primers to detect levels of plant nuclease I (pollen extracellular nuclease) were derived in this study and designated as *Nt*PNI/53 °C (Table S1). Results were normalized to actin as internal control using *Nt*Actin/51 °C.

Two markers of senescence were analyzed: “no apical meristem, ATAF1/2, cup-shaped cotyledon2” (NAC) *Nt*NAC080 TF, a positive regulator of senescence, and senescence-specific cysteine protease (*Nt*CP1) [24]. These markers were amplified with primers *Nt*NAC/60 °C and *Nt*CP1/60 °C, respectively, together with actin using primers *Nt*ACT2/60 °C (Table S1) [24].

RT-qPCR was run on the CFX Connect™ Real-Time device (Bio-Rad) using 20 μL reaction mixture containing 5 μL 35× diluted cDNA, 5 μL 2 μM forward and reverse gene-specific primers (Table S1) and 10 μL 2× SYBR™ Green PCR master mix (Applied Biosystems, Waltham, MA, USA), under the following amplification condition: initial denaturation at 95 °C for 3 min, followed by 40 cycles of denaturation at 95 °C for 30 s, annealing at temperatures as above for 30 s and extension at 72 °C for 35 s. At the end of the reaction, the specificity of each primer pair was assessed using a melting curve analysis. Product sizes were confirmed by melting analysis and 2% agarose gel electrophoresis. The abundance of *Nt*actin as a reference was estimated in parallel in each sample. Ct values were measured using CFX Maestro qPCR software v.1.1 (Bio-Rad). The relative values were standardized with the delta-delta method and normalized to the sample with germinating pollen, where the calibrator was set to 100%. The data points show the means ± SD of two replicates of each PCR reaction and calculated statistic results are marked in individual figure legends.

### 2.4. Other Methods

The cDNA libraries were prepared and next generation sequencing (NGS) was performed by Macrogen Europe BV (Amsterdam, The Netherlands) using RNA isolated and purified using the same procedure as for mRNA quantification. NGS was performed using Illumina SBS technology. The duplicated NGS samples contained 5.9 and 7.4 GB bp having 39 × 10^6^ and 49 × 10^6^ reads, respectively. The raw sequencing data was trimmed using Trimmomatic v0.30 [25].

Sequence comparisons were carried out with DNASIS v2.6 (Hitachi Software Engineering Company, Tokyo, Japan). Protein domain sequence analyses were performed using the module InterProScan of Geneious Prime® 2022.0.1; the same software using option “Align/Assemble map to reference” was used for NGS reads mapping to TFIIIA-9ZF and TFIIIA-7ZF target. For the analysis of performance parameters of *Nb*TFIIIA-7ZF transgenotes of *N. tabacum* individual samples were photographed and then analyzed with the “measurement” option using LUCIA v.5.0 software (Laboratory Imaging, Prague, Czech Republic).

## 3. Results and Discussion

### 3.1. The Ectopic Expression of TFIIIA-7ZF, the Splicing Variant of Viroid-Modulated TFIIIA, Causes Morphological Irregularities in Transgenic N. tabacum

**Figure 1:**
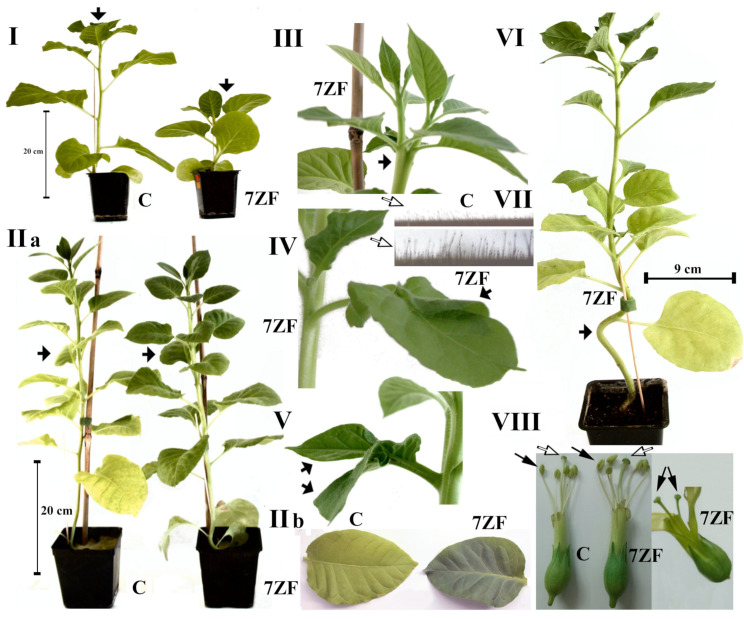
Growth characteristics and multiple morphological deviations of *Nicotiana tabacum* observed in plant lines transformed with viroid-induced factor TFIIIA-7ZF driven from chalconsynthase promoter Pchs_H1 (Figure S2). Changes in TFIIIA-7ZF transformed plants (7ZF) versus untransformed controls (C) are indicated. The figure shows retardation of growth and severe stunting of young plants (**I**); delay of senescence and aging (**IIa**) which is manifested by chlorophyll decomposition and disappearance, and by a change to yellow coloration of leaves (**IIb**); splitting of apexes causing branching of the main plant apex as indicated by the arrow in panel (**III**); by bifurcating of leaf blades in the upper part of the leaves as shown by the arrow in panel (**IV**) or by splitting of leaf petioles causing formation of double-blade leaves (**V**); by deformation of main stems in lower parts (**VI**); increasing of trichome length and sizes forming hairy stems (**VII**); enlargement of anthers (black arrows) and pistils (empty arrows) in flowers of 7ZF plants and seldom splitting of pistils as indicated by the double arrow in panel (**VIII**).

In the present study we used the plant transformation vector bearing *Nb*TFIIIA-7ZF (see Figure S1b) cDNA driven by chalcone synthase chs_H1 from hop [26] (Figure S2) to analyze ectopic overexpression of the *Nb*TFIIIA-7ZF transgene in *N. tabacum*. Transformed tobacco regenerates grew vigorously on media containing kanamycin; in vivo lines expressed GFP signal under UV at 301nm. A significant level of kanamycin resistance gene NPTII mRNA was observed by RT-qPCR (Figure S3a). A statistically significant increase of TFIIIA levels in 7ZF tobacco leaves was measured in comparison to control untransformed tobacco (Figure S3a). Plants transformed with *Nb*TFIIIA-7ZF surprisingly showed numerous morphological deviations and abnormalities (Figure 1), which were not observed in control tobaccos. Especially severe plant stunting was observed (Figure 1I), splitting apexes and irregular branching of shoots (Figure 1III), formation of double-bladed leaves (Figure 1IV), splitting of leaf petioles (Figure 1V), deformation of main stems (Figure 1VI), and modification of glandular trichomes (Figure 1VII), which were bigger and longer in 7ZF transgenotes. The glandular trichomes on the main stem of 7ZF were stronger and longer by 161% then that of controls (100%). Other deviations included doubling of pistils (Figure 1VIII, double arrows), or bigger anthers and pistils in 7ZF plants than in controls (Figure 1VIII, compare flower C and 7ZF). In addition, rough leaf edges were observed in 7ZF transgenotes, while leaf edges were quite smooth in untransformed controls (not shown).

### 3.2. Delayed Aging and Senescence in TFIIIA-7ZF Transgenotes of Tobacco

In addition to morphological changes and a significant delay in plant development, later flowering was observed in case of 7ZF transformants compared to controls. Furthermore, aging and senescence were delayed in 7ZF plants (Figure 1IIa). The senescence was easily visible on leaves due to chlorophyll decomposition and disappearance (Figure 1IIb), and by a change to yellow coloration of leaves (Figure 1IIb). During this interval, 7ZF plants showed dark green coloration, although still stunted for approximately 20% in comparison to controls. Leaves collected from the upper third of stems showed only 75% integrated green color density of that in 7ZF transgenotes (100%), according to comparative measurements by the image analyzer.

**Figure 2:**
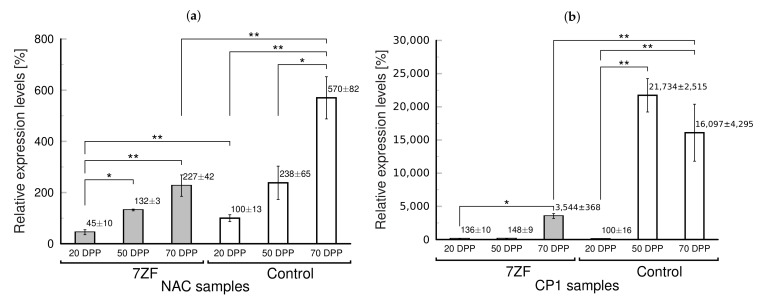
Relative levels of mRNA of two markers of senescence in control and *Nb*TFIIIA-7ZF (7ZF)-transformed tobacco. Two factors analyzed by Li et al. [24] were quantified by RT-qPCR to characterize tobacco senescence on molecular level in 7ZF and control leaves collected at 20, 50, and 70 days of seedling post pricking out (DPP). (**a**) TF *Nt*NAC080 (NAC), which was described as regulator of scenescence [24] and (**b**) senescence marker *Nt*CP1 (CP1). Tobacco leaves collected from the upper third of plant shoots were extracted and analyzed as described in Materials and Methods. Expression levels were normalized to actin. The levels in controls 20 DPP were taken as 100%. The mean values ± SD of two replicates of each PCR reaction are shown. Lines with asterisks indicate statistically evaluated differences between connected values (*, statistically non-significant differences at *p* > 0.05; **, statistically significant differences at *p* < 0.05).

We used two senescence factors of tobacco [24] to assay the level of senescence on the molecular level (Figure 2). NAC080 is member of a TF family regulating leaf senescence in tobacco, while CP1 is used as marker of senescence [24]. It is seen that NAC mRNA is gradually increasing over time, but in controls the increase is shifted to higher levels (Figure 2a). CP1 levels were low and not different in young plants, but there was a strong increase detectable in controls with a maximum at 50 days of cultivation (Figure 2b). Thus, the changed leaf coloration, a possible initial symptom of aging, was in accordance with strong accumulation of senescence markers in control leaves (Figure 2) supporting that the 7ZF transgene significantly delayed the senescence.

### 3.3. Transient Somatic Depression of Selected “Degradome” Due to *Nb*TFIIIA-7ZF Overexpression

In principle, plant nucleases could be involved in delayed senescence and in some morphological changes like bending of stems observed in 7ZF-transgenic tobacco. We observed similar morphological irregularities and also a significant delay of senescence when we used virus-induced gene silencing (VIGS) to suppress expression of tomato and *Arabis brassica* multifunctional nuclease I (unpublished). This major plant nuclease (PNI) (Figure S1a) was characterized by crystallography in our previous work [27]. It is a zinc-dependent acid endonuclease non-specific for the sugar component of nucleic acids. PNI has the capability to cleave even thermodynamically stable RNAs like double-stranded RNA, 7SL RNA, viroid RNA, and single- or double-stranded DNA. In addition it is exhibiting 5’ nucleotidase and phospholipase activity [27,28]. These plant enzymes are well described to play an important role in formation of xylem tissue by an apoptotic process [29]. PNI is involved in plant aging and senescence (e. g., [30,31]). PNI was observed in our previous work to be developmentally overexpressed in maturing anthers [32,33] and therefore, we added this enzyme to our concept of “degradation complex”. We already described that this enzyme mainly has pollen extracellular activity and we hypothesized that it could play some role in pollen nutrition by cleavage of nucleic acids from apoptotic, degenerated anther tissues and tapetum layer during pollen development [32]. It was originally found that the nuclease can be washed out from mature and germinating tobacco pollen [34] and that enzyme activity is practically not detectable in mature tobacco pollen [32]. Although this activity is negligible in mature and germinating pollen, it could be active inside of immature pollen or could function as some barrier against pathogenic RNAs in anthers. In this respect some question still remained about mRNA levels of PNI inside immature pollen at early stages (stage 3), where the enzyme shows peak activity in whole anthers. Our RT-qPCR analysis (Figure S4) shows that PNI mRNA levels are highest in whole anthers (Figure S4, A3). It was still detectable but at 23× lower level inside developing pollen on stage P3 in comparison to whole anthers (Figure S4). The level was, however, still roughly about 20× higher in P3 than in mature pollen. This suggests possible nuclease biosynthesis inside pollen on very early stages, and transportation to the microspore surface. Alternatively, there is a dual origin of PNI from pollen and anther tissues. Level of PNI mRNA is very negligible from stage P5 (55%) and not significantly different from mature and germinating pollen (Figure S4). These results are consistent with previous experiments performed using detection of enzymatic activity of PNI in anthers during pollen development [32,33]. For comparison, we plotted relative levels of other analyzed degradome components at stage P5 in Figure S4. The highest level was detected for TSN (8900% compared to PNI in pollen tubes as control, 100%), then DCL (1800%) and the lowest level for AGO5 (188%) (Figure S4). AGO5-mediated viroid cleavage during development of tobacco pollen was, however, clearly demonstrated in our previous work [11], suggesting that this mRNA is not abundant during pollen development, but the factor is very specific for the process of viroid elimination from pollen.

**Figure 3:**
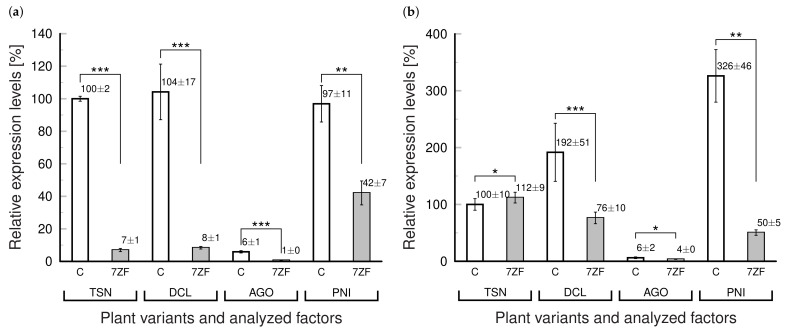
Relative mRNA levels of factors forming potential viroid “degradation complex” in *N. tabacum* transformed with NbTFIIIA-7ZF. Relative mRNA expression levels of factors previously analyzed in developing pollen [11] *Nt*AGO5 (AGO), *Nt*DCL (DCL) and *Nt*Tudor S-like nuclease (TSN) and pollen extracellular nuclease I (PNI) (see Figure S4) were assayed using RT-qPCR in tobacco leaves collected from the upper third of plant shoots 20 (**a**) and 50 days (**b**) after seedling pricking out and subsequent cultivation. Untransformed plants were used as controls (C) to *Nb*TFIIIA-7ZF transgenotes (7ZF). Results of RT-qPCR performed as described in Materials and Methods were normalized to actin. The level of AGO mRNA in control tobacco leaves was taken as 100%. The mean values ± SD of two replicates of each PCR reaction are given. Lines with asterisks indicate statistically evaluated differences between connected values (*, statistically non-significant differences at *p* > 0.05; **, statistically significant differences at *p* < 0.05; ***, statistically significant differences at *p* < 0.01).

In further experiments the levels of PNI and other components of selected “degradation complex” (TSN, DCL and AGO) potentially important for viroid propagation in anthers [11] were quantified in leaves of 7ZF transgenotes (Figure 3). Surprisingly, levels in 7ZF plants, 20 days after transfer from the pricking device to pots, showed significantly lower values for all degradation factors (Figure 3a) suggesting significant influence of 7ZF transgene on the analyzed degradation complex. For instance, TSN mRNA was about 14×, DCL about 12×, AGO 8×, and PEN 2× lower in 7ZF transgenotes than in controls. However, this relative expression changed with plant age. Leaves sampled one month later showed no difference in TSN. Some decrease in the mean values of relative mRNA levels for DCL and AGO were detected, showing similar tendency in suppression of these factors as in young plants; these differences, however, were not statistically significant (Figure 3b). A significant difference at this interval was observed between PNI levels in control and 7ZF plants (Figure 3b), where PNI showed 6.5× lower level in 7ZF than in controls, suggesting a rather strong depression of this degradation factor.

We conclude, based on comparison of Figure 2 and Figure 3, that the “misregulation” caused by the 7ZF transgene is interfering at later stages with the plant senescence network. The comparisons during continuous plant cultivation period showed that there is the strongest depression especially of PNI nuclease in 7ZF plants, while mRNA levels of TSN, DCL, and AGO significantly increased with plant aging (compare Figure 2 and Figure 3). While controls showed a dramatic increase of PNI together with the initial symptoms of aging (changed leaf coloration, increasing levels of CP1 and NAC), transgenes 7ZF were without obvious symptoms and kept relatively low PNI levels.

### 3.4. The “Degradation Complex” upon Viroid Infection in Tobacco Transformed with Viroid cDNAs and Potential Role of TFIIIA-7ZF in Viroid Adaptation and Pathogenesis

**Figure 4:**
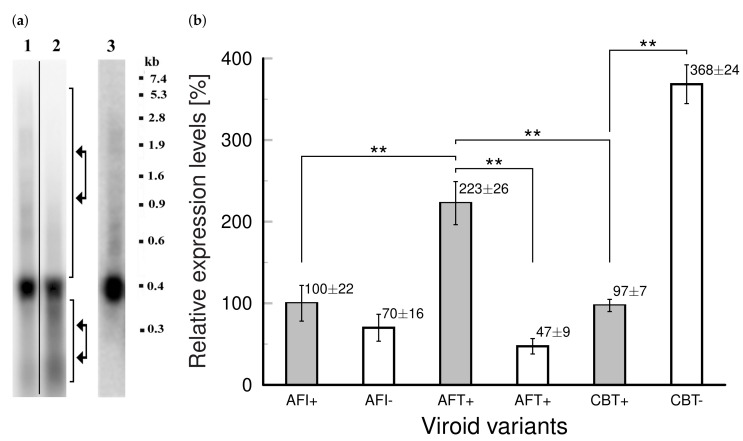
Northern blot analysis of AFCVd intermediates in infected and transformed tobacco (**a**) and quantification of AFCVd and CBCVd in infected/transformed tobacco leaves by qPCR (b). (**a**) Northern blot analysis was performed using 35 µg of total RNA per lane from leaf tissue collected from plants approximately 60 dpi or 70 days after growing seedlings from transformant’ seeds. Lane 1, sample from young upper leaves (third position from the stem apex) of AFCVd-infected plants. Lane 2, sample from leaves showing the beginning of scenescence (ninth position from stem apex). Lane 3, sample from young upper leaves (third position from the stem apex) of AFCVd-transformed/infected plants. Samples on lane 1 and 2 were run on the same gel. Samples were probed by [32P-dCTP]-labeled viroid cDNA as described in Materials and Methods. Membranes were scanned after 24 h exposure. Arrows in lane 2 indicate detectable breakdown fragments shorter than unit-length monomeric viroid RNA and a more light zone corresponding to signals of replication intermediates in older leaves. The marker ladder on the left is the ethidium bromide-stained RNA III marker (Boehringer Mannheim). (**b**) Relative levels of viroid (+) and (−) RNA chains in young upper leaves of infected (AFI) and transformed/infected plants with AFCVd (AFT) and in transformed/infected plants with CBCVd (CBT) after 40 days of cultivation. The levels of AFI+ chains are taken as 100%. Relative expression levels were normalized to 7SL RNA. Columns represent the mean ± SD of two replicates of each PCR reaction. All statistically evaluated differences shown by columns connected by lines with double asterisks were statistically significant at *p* < 0.05.

It has been shown by Wang et al. [13] that suppression of TFIIIA-7ZF reduced PSTVd replication, and overexpression of TFIIIA-7ZF enhanced PSTVd replication in planta in leaves of *N. benthamiana*. Based on these recent reports supporting the involvement of modified TFIIIA in viroid propagation [13,14], together with the results presented in this study about 7ZF-mediated transient suppression of analyzed degradation complex, we aimed to assay expression of the analyzed degradome under viroid infection. With AFCVd, which can infect tobacco as RNA after biolistic inoculation [11], no significant viroid degradation was detected in young leaves using classical Northern blot, while in leaves at the beginning of senescence a low level of longer-than-unit-length (oligomeric) intermediates and also accumulation of shorter-than-unit-length cleavage products were detectable (Figure 4a). This confirms that this viroid is degraded during the propagation more significantly in older leaves and some cleaving products of this degradation are quite visible, similarly as detected during the maturation of pollen [11]. This means, viroid is degraded during its propagation in somatic leaf tissues by a spectrum of unspecific and specific nucleolytic activities. It has to be noted that the DICING process is hardly visible on the classical Northern blot presented in Figure 4a. However, accumulation of viroid small RNAs even in young leaves was described in our previous experiments [15] and in many analyses performed by others (for review see e.g., [35,36]). A viroid degradation pathway in somatic tissues involving endonucleolytic cleavage was described in the work of [37]. The predominant cleavage in somatic tissues produced 5’OH and 3’ P termini [37]. This could be caused by some endoribonucleases like mutifunctional TSN (for review see [38]) and/or E-like endoRNases or endoRNases from the T2 family, which are detectable in pollen [33], but were not included in our degradation complex because there were only non-significant differences in expression profiles of healthy and viroid-infected developing pollen [11]. However, we cannot exclude that E-like or T2-like RNAses do participate in viroid cleavage in some particular stage of plant development or tissue(s). The same is true for PNI capable of degrading very stable RNA structures including viroid [28], especially in tissues like anthers where its very abundant. Note, however, that this enzyme is producing 5’ P and 3’ OH ends, which were found to be not dominant [37], but can, in principle, be re-ligated by the viroid-redirected DNA ligase [39].

To initiate infection of CBCVd it was necessary to transform tobacco with viroid cDNA [11]. We used specific pFAST vectors (Figure S5) bearing dimeric viroid sequences under a pollen promoter that has negligible activity in somatic tissues, but efficiently promotes infection in plants. Two vectors (Figure S5) bearing pollen non-transmissible viroid cDNAs of interest, AFCVd (AF vector) and CBCVd (CB vector) [11,12], respectively, were used in parallel to biolistic AFCVd infection to prepare viroid transformed/infected tobaccos. AF and CB transformants expressed GFP in the leaves and NPTII gene for resistance to kanamycin (Figure S3b).

Quantitative assays using singlestrand-specific RT-qPCR (Figure 4b) showed significantly higher level of AFCVd in upper young leaves of AFCVd transformed/infected plants than in leaves in the same position of untransformed, biolistically inoculated AFCVd-infected plants (Figure 4a,b). Because of negligible activity of selected pollen promoter in somatic tissues, this increase can be most probably attributed to transformation/infection of the whole cellular pool in leaves; this could represent the major difference from untransformed inoculated/infected tissues. Transformation/infection of tobacco also promoted high level of CBCVd with significant prevalence of (−) chains (Figure 4b), which is consistent with our previous results [11,20]. Both hop viroid isolates showed strong symptoms in infected and especially in transformed/infected *N. benthamiana* plants with symptoms like drastic stunting, tiny leaves, and production of defective pollen [12]. In contrast, very mild or no symptoms were visible on infected/transformed shoots and leaves of *N. tabacum*. This enabled us to analyze levels of the “degradation complex” with lower probability of the presence of strong pathogenic network(s), which would complicate this analysis. We knew for instance from our previous studies that in symptomatic leaves of tomato some regulatory factors like SANT/HTH Myb were strongly depressed due to viroid [40]. This premature depression by PSTVd was similar to depression induced by the scenescence network [40]. Also the tomato homologue of PNI was enhanced in symptomatic leaves and tissues in comparison to controls [15].

**Figure 5:**
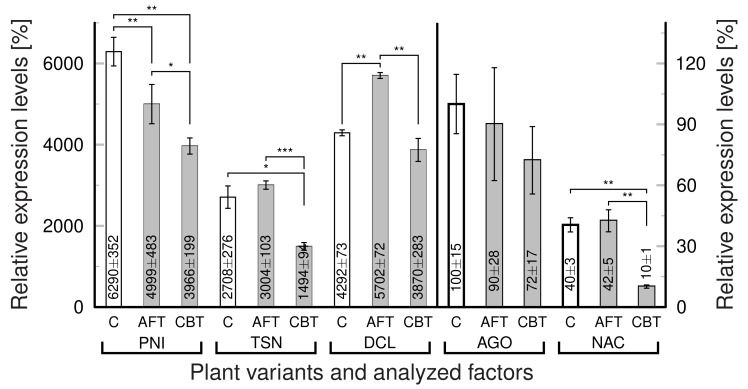
Relative expression levels of the “degradation complex” in control and viroid-infected tobacco leaves. The set of factors that could participate in viroid degradation is the same as described in the legend of Figure 3. In addition to the “degradation complex” samples were quantified for TF *Nt*NAC080 (NAC) involved in the regulation of senescence in tobacco [24]. Young upper leaves were collected from control (C), AFCVd transformed/infected (AFT) and CBCVd transformed/infected (CBT) plants in the stage of early flowering, RNA was extracted and subjected to RT-qPCR analysis as described in Material and Methods. Expression levels were normalized to actin. The levels of AGO in the controls were taken as 100%. The mean values ± SD of two replicates of each PCR reaction is given. Lines with asterisks indicate statistically evaluated differences between selected variants (*, statistically non-significant differences at *p* > 0.05; **, statistically significant differences at *p* < 0.05; ***, statistically significant differences at *p* < 0.01).

Analysis of relative mRNA levels of the “degradome” in young upper leaves of AFCVd and CBCVd transformants showed some differences between these variants, as well as differences to control (Figure 5). A statistically significant decrease of PNI in comparison to controls was found for AFCVd transformants, significantly lower levels of PNI and TSN compared to controls were observed for CBCVd transformants. DCL mRNA in AFCVd transformants increased and differed from both, controls and CBCVd transformants. No statistically significant differences were found for AGO among analyzed variants. Moreover, AFCVd transformants showed a similar level of NAC to control, while CBCVd transformants exhibited lower level of this factor and significantly differed from both these variants. This suggests some shift in the “aging status” in CBCVd transformants in comparison to AFCVd and control. In fact, there were some differences observed in flowering dynamics in CBCVd transgenotes in comparison to AFCVd and controls. In all these analyzed variants we quantified TFIIIA mRNA levels in the same RNA extracts (Figure S3). RT-qPCR analysis showed enhanced levels of TFIIIA, but no statistically significant differences between AFCVd and CBCVd transgenotes or in comparison to control.

For the AFCVd transgenote, we performed a supplementary analysis by mapping NGS reads to TFIIIA-7ZF and -9ZF mRNAs, as shown in the scheme depicted in Figure S1c. The mapping of high identity reads from AFCVd transgenotes showed an unequal distribution between these two TFIIIA variants of different lengths. Density of reads for TFIIIA-9ZF mRNA was 4.38 reads per bp (R/bp) while it was 5.04 R/bp for TFIIIA-7ZF. In principle, the distribution calculated for TFIIIA-9ZF should give about 3771 reads covering the TFIIIA-7ZF fragment; however, there was a discrepancy of 569 reads or 11.6% in favor of TFIIIA-7ZF. This suggests a higher fraction of TFIIIA-7ZF then -9ZF in AFCVd-transformed/infected tobacco leaves.

TFIIIA-7ZF is generated upon viroid infection by alternative splicing [14] and is involved in viroid propagation and replication pathway driven by plant DNA-dependent RNA polymerase II [13,14]. In regulation of TFIIIA-7ZF biogenesis another factor is involved, ribosomal protein L5 (RPL5) [41], which acts as a negative regulator of this process and plays important role together with TFIIIA in 5S rRNA biogenesis [7]. We have not analyzed this particular regulatory pathway in connection to TFIIIA-7ZF, but we showed another feature of TFIIIA-7ZF if ectopically overexpressed in tobacco. According to our results this modified factor can cause transient depression of “degradome” during plant development. In addition, we showed that this process obviously interferes with plant aging and senescence, which was significantly delayed by the TFIIIA-7ZF transgene. As a concomitant phenomenon of the TFIIIA-7ZF network, there are some physiological and morphological irregularities and deviations in transgenic *N. tabacum*. In our previous report we provided supplementary data showing that TFIIIA-7ZF has the ability to interfere with various transcription complexes in the transient expression system. For instance, TFIIIA-7ZF co-expression in agroinfiltrated *N. benthamiana* leaves significantly influenced the tripartite Myb2/bHLH2/WDR1 (MBW) [26] and bipartite WRKY1/WDR1 (WW) [42] regulatory complexes, suggesting its potential to modulate or change expression of some genes [11]. This could be the reason for plant morphogenesis modifications upon overexpression of TFIIIA-7ZF and more complex interactions during plant senescence.

Based on quantifications of TFIIIA and RPL5 mRNAs in developing pollen, we proposed in our previous work that the status of abundant proteosynthesis machinery in pollen could lead to a lack of TFIIIA-7ZF [11] in this tissue with the consequence of low rate of viroid propagation and, finally, to its complete elimination. This complete viroid elimination is due to co-expression of a set of factors forming an active degradation complex, which operates in maturing and germinating pollen [11]. Based on the presented results, we can assume that, in addition to a lack of viroid replication, the low level of TFIIIA-7ZF could maintain high levels of the RNA degrading activities in developing pollen, supporting the mechanism of viroid elimination and confirming the potential role of TFIIIA-7ZF in this process. In our previous work, we showed severe pathogenic effects of pollen- and seed-non-transmissible AFCVd and CBCVd on transformed/infected *N. benthamiana* pollen and we predicted some level of adaptation of pollen-transmissible viroids to pollen metabolism [12]. It is not known, however, whether TFIIIA-7ZF could play some role in this adaptability and/or rather some viroid protection factors play the major role in the viroid transmissibility, as we speculated previously [11,15].

Tobacco belongs to rather symptomless plants; no significant symptoms appear when infected with analyzed members of family *Pospiviroidae*, AFCVd and CBCVd. We produced transformed plants bearing viroid cDNAs to initiate efficient propagation of AFCVd and CBCVd in tobacco leaf tissues and cells. Variable results on “degradome” profile in transformed/infected leaves suggests that the phenomenon is physiologically very complex and depends on viroid species. In general, degradome levels were more depressed in leaves infected with CBCVd than with AFCVd. These results are in the context to other studies showing complex impact and/or adaptation of various viroids to different plant hosts. Differences between AFCVd and CBCVd in expression of WRKY and prenylflavonoid biosynthesis genes were found in hop *Humulus lupulus* [20] as well as in the case of some ribosomal genes on symptomatic *N. benthamiana* [12]. It is not known in this respect, whether an involvement of TFIIIA-7ZF in viroid propagation/pathogenesis and plant morphogenesis represents a more general phenomenon. At least, a recent report about its potential role in hop latent viroid-infected hop [43] and our unpublished results about some morphological changes mediated by hop-specific *Hl*TFIIIA-7ZF in tobacco should support further physiological studies of this factor.

## Data Availability

Not applicable.

## Supplementary Materials

The following are available online at https://www.mdpi.com/article/10.3390/cells11050784/s1. Supplementary figures: Figure S1. Protein domains of *Nt*PNI, *Nb*TFIIIA-7ZF, and *Nt*TFIIIA. Figure S2. Schematic representation of the plant vector pJM14 with integrated *Nb*TFIIIA-7Z. Figure S3. Relative levels of TFIIIA and NPTII mRNAs in tobacco plants transformed either with vector pJM14 containing *Nb*TFIIIA-7ZF or with infectious pFAST vectors bearing dimeric AFCVd or CBCVd cDNAs. Figure S4. Relative mRNA levels of pollen extracellular nuclease (PNI) mRNA in various stages of tobacco anthers and pollen development Figure S5. Plant vector cassettes used for transformation of *N. tabacum* with AFCVd and CBCVd infectious dimeric (++) cDNAs. Supplementary table: Table S1. Primers used for strand-specific RT-qPCR and quantification of mRNA levels by RT-qPCR.
