# Supplementary material for: The Splicing Variant TFIIIA-7ZF of Viroid-Modulated Transcription Factor IIIA Causes Physiological Irregularities in Transgenic Tobacco and Transient Somatic Depression of “Degradome” Characteristic for Developing Pollen
Source: Cells. 2022 Feb 23;11(5):784. doi: 10.3390/cells11050784 (PMC8909551; doi:10.3390/cells11050784)
Supplement: Supplementary file 1 [file cells-11-00784-s001.zip › cells-1583640-supplementary.pdf]

Supplementary Materials: The splicing variant TFIIIA-7ZF of viroid-modulated transcription factor IIIA causes physiological irregularities in transgenic tobacco and transient somatic depression of "degradome" characteristic for developing pollen

Jaroslav Matoušek [1] and Gerhard Steger [2,*]

List of Figures

S1. Protein domains of *Nt*PNI, *Nb*TFIIIA-7ZF, and *Nt*TFIIIA 2
S2. Schematic representation of the plant vector pJM14 with integrated *Nb*TFIIIA-7Z 3
S3. Relative levels of TFIIIA and NPTII mRNAs in tobacco plants transformed either with vector pJM14 containing *Nb*TFIIIA-7ZF or with infectious pFAST vectors bearing dimeric AFCVd or CBCVd cDNAs 4
S4. Relative mRNA levels of pollen extracellular nuclease (PNI) mRNA in various stages of tobacco anthers and pollen development 5
S5. Plant vector cassettes used for transformation of *N. tabacum* with AFCVd and CBCVd infectious dimeric (++) cDNAs 6

List of Tables

S1. Primers used for strand-specific RT-qPCR and quantification of mRNA levels by RT-qPCR 7

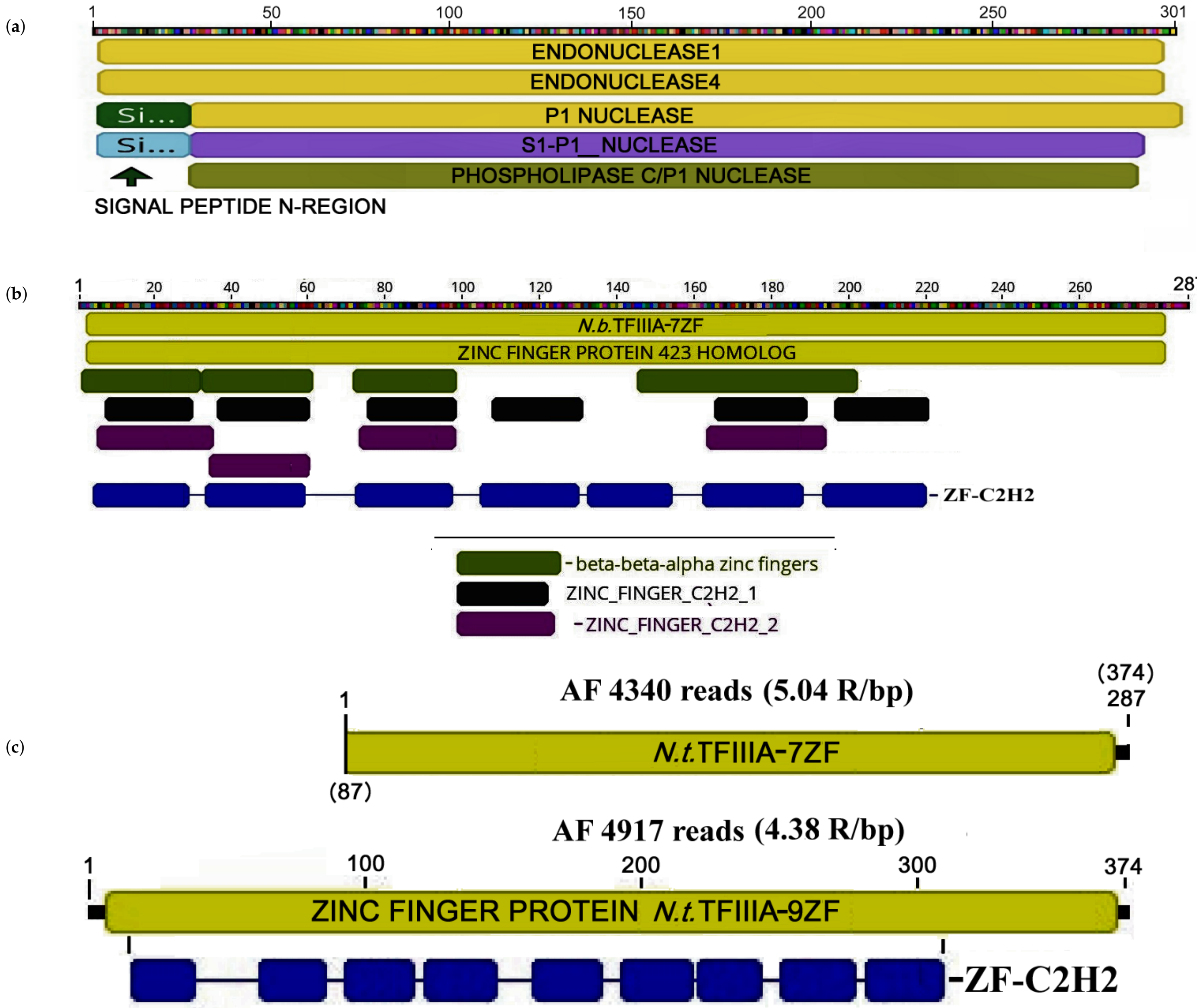

Figure S1. Protein domains of (a) *Nt*PNI (pollen extracellular nuclease I, plant nuclease I, GenBank AC XP_016459651.1), (b) *Nb*TFIIIA-7ZF [1], and (c) *Nt*TFIIIA. Domains were predicted and visualized using InterProScan [2] and Geneious Prime 2020.04 software, respectively.

(a) PNI is a potential member of pollen "degradation complex" supplementing our analyzes performed previously [1].

(b) *Nb*TFIIIA was amplified from *N. benthamiana* plants infected with lethal PSTVd strain AS1 [3]. This authentic sequence was cloned into vector pJM14 [1] and transformed in this study to *N. tabacum*. *Nt*TFIIIA-9ZF homologue was derived from *N. tabacum* genomic sequences using BLAST [4].

(c) *Nt*TFIIIA-9ZF cDNA (top) and the corresponding nucleotide fragment covering *Nt*TFIIIA-7ZF (bottom) cDNAs were used as targets for mapping of high identity NGS reads from AFCVd transformed/infected tobacco using Geneious Prime® 2022.0.1 (see Material and Methods). The total number of reads per sequenced transcriptome and the density of corresponding reads per bp of cDNA fragment (R/bp) are indicated above the aa sequence schemes.

(**a**) (**b**)

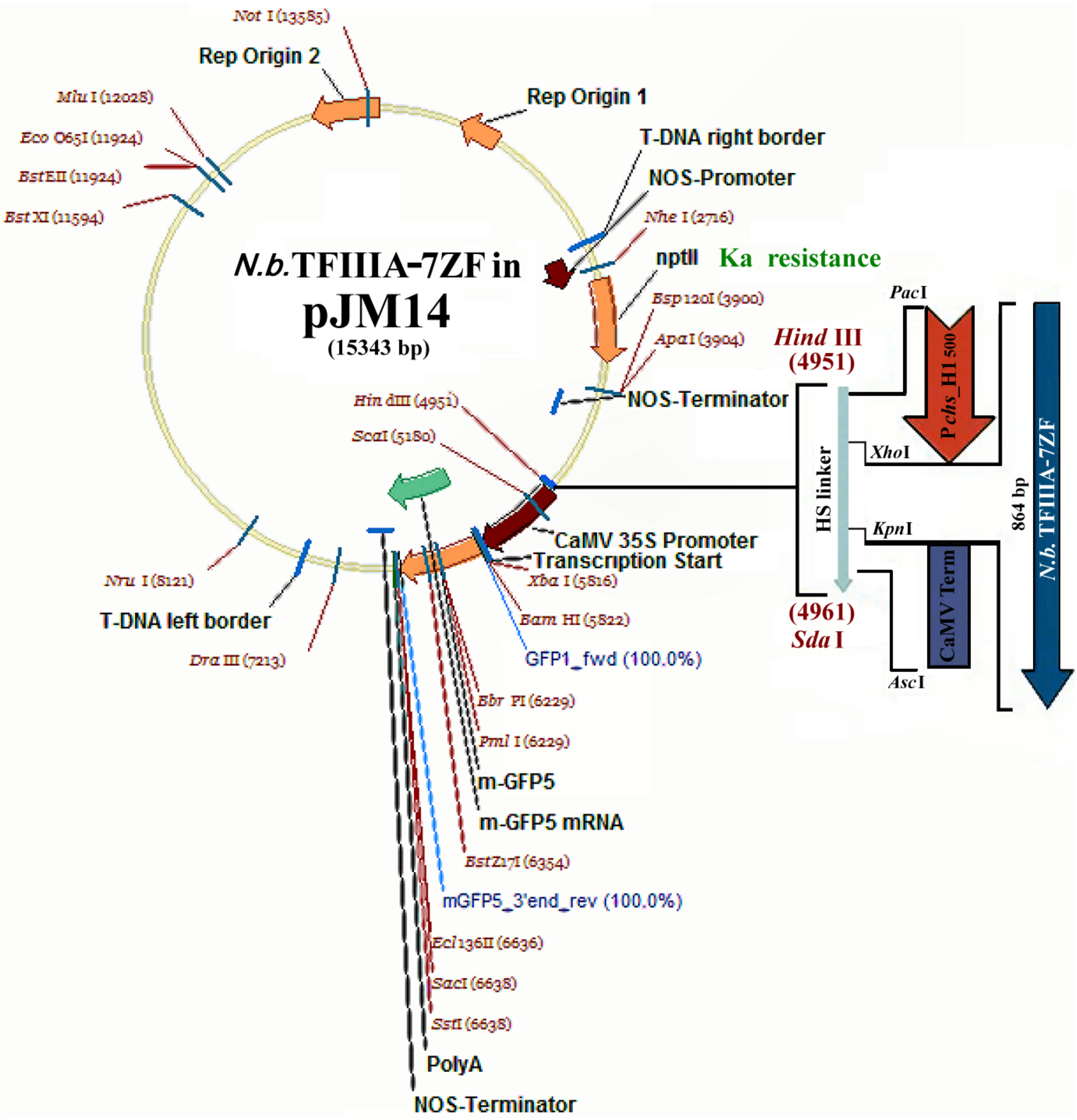

Figure S2. Schematic representation of the plant vector pJM14 with integrated *Nb*TFIIIA-7Z. (**a**) The *Nb*TFIII-7Z cDNA (864 bp) was integrated into pJM14 [1] using the unique restriction sites *Xho*I and *Kpn*I within the sequence of the expression cassette (**b**). The scheme in (**b**) is not to scale. The vector provides plant resistance to kanamycin.

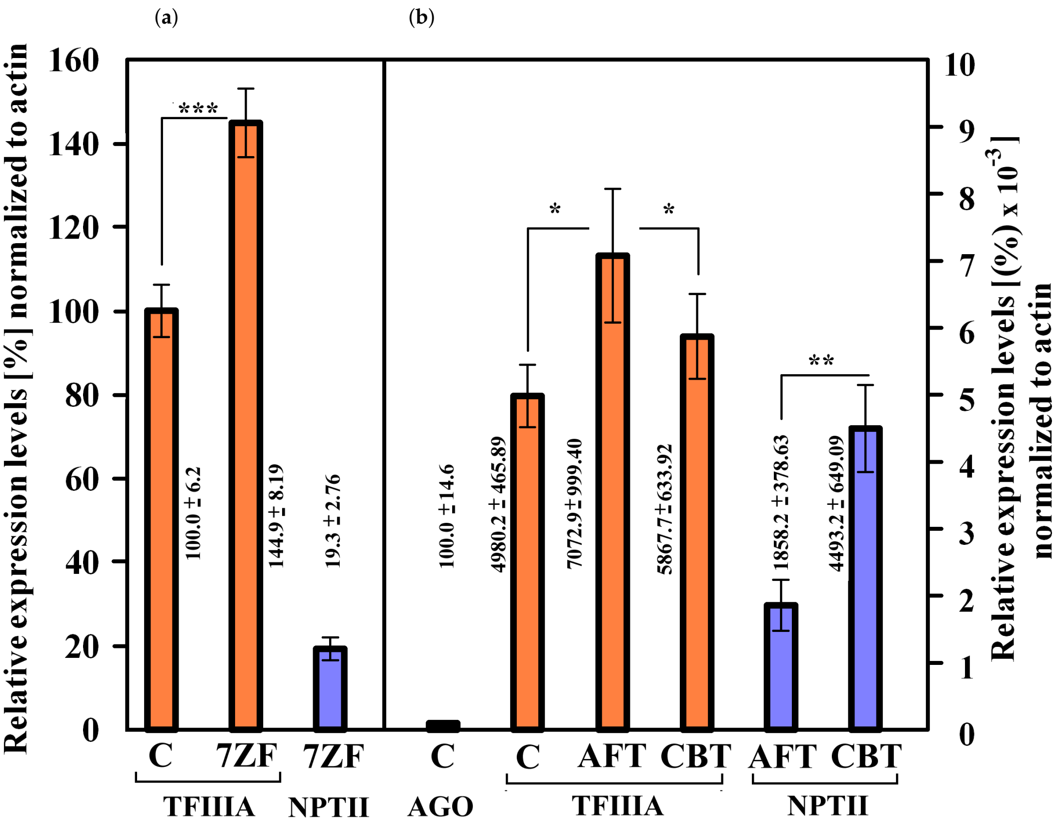

Figure S3. Relative levels of TFIIIA and NPTII mRNAs in tobacco plants transformed either with vector pJM14 containing *Nb*TFIIIA-7ZF (**a**) or with infectious pFAST vectors (see Figure S5) bearing dimeric AFCVd (AFT) or CBCVd (CBT) cDNAs (**b**).

(**a**) Level of endogenous TFIIIA in untransformed control (C) is shown, while the level in transformants (7ZF) includes endogenous TFIIIA together with transgene-expressed *Nb*TFIIIA-7ZF (orange columns). For transgenic plants 7ZF also level of mRNA for kanamycin resistence gene (NPTII) is shown (blue column). No specific NPTII signals were observed for control tobacco plants.

(**b**) Levels of endogenous TFIIIA are shown for control (C) and transgenic AFT and CBT variants (orange columns). Levels of NPTII mRNA in transgenotes are shown by the blue columns. No specific NPTII signal was detectable in controls. Each column represents the mean ± SD of two replicates of each PCR reaction. The level of AGO in controls was set to 100%.

Lines with asterisks indicate statistical differences between connected values (*, statistically non-significant differences at $p > 0.05$; **, statistically significant differences at $p < 0.05$; ***, statistically significant differences at $p < 0.01$).

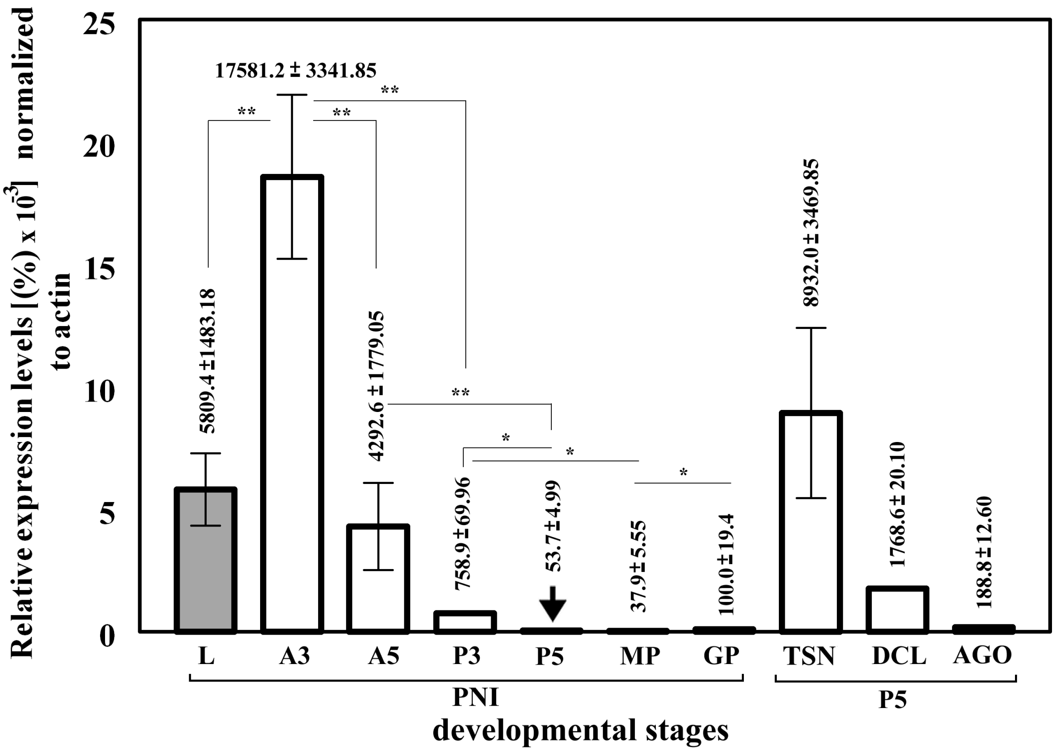

Figure S4. Relative mRNA levels of pollen extracellular nuclease (PNI) mRNA in various stages of tobacco anthers and pollen development. For comparison, PNI levels are shown in young leaves (L), in whole anthers of developmental stages 3 (A3) and 5 (A5), respectively, in isolated immature pollen of stages 3 (P3) and 5 (P5), respectively, in mature pollen (MP), and in 6 hours germinating tobacco pollen (pollen tubes, GP). The levels of PNI in GP was taken as 100%. The asterisk indicates very low level of PNI already in stage P5. In the right part of the graph, comparative analysis of the levels of other factors that could participate in viroid degradation in pollen [1] are shown at critical stage P5 of pollen development: *Nt*Tudor S-like nuclease (TSN), *Nt*DCL (DCL), and *Nt*AGO5 (AGO). Results of qPCR, performed as described in Materials and Methods, were normalized to actin. The mean value ± SD of two replicates of each PCR reaction is shown. Lines with asterisks indicate statistically evaluated differences between connected PNI values (*, statistically non-significant differences at $p > 0.05$; **, statistically significant differences at $p < 0.05$; ***, statistically significant differences at $p < 0.01$).

(**a**)

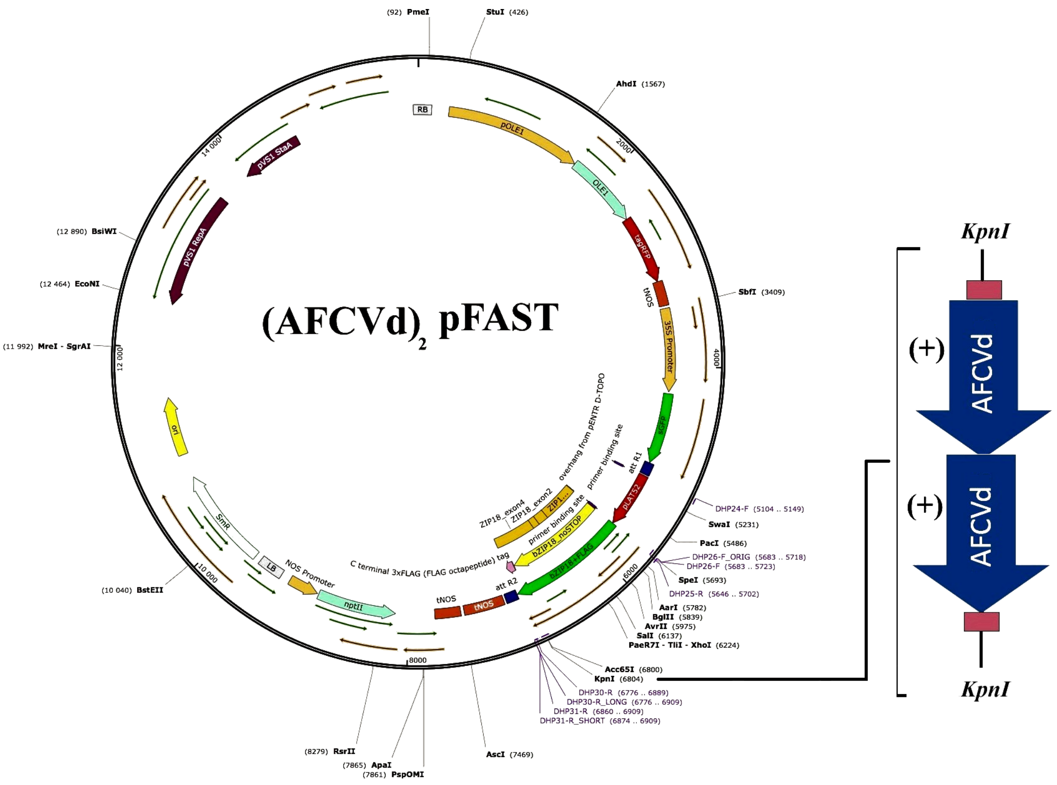

(**b**)

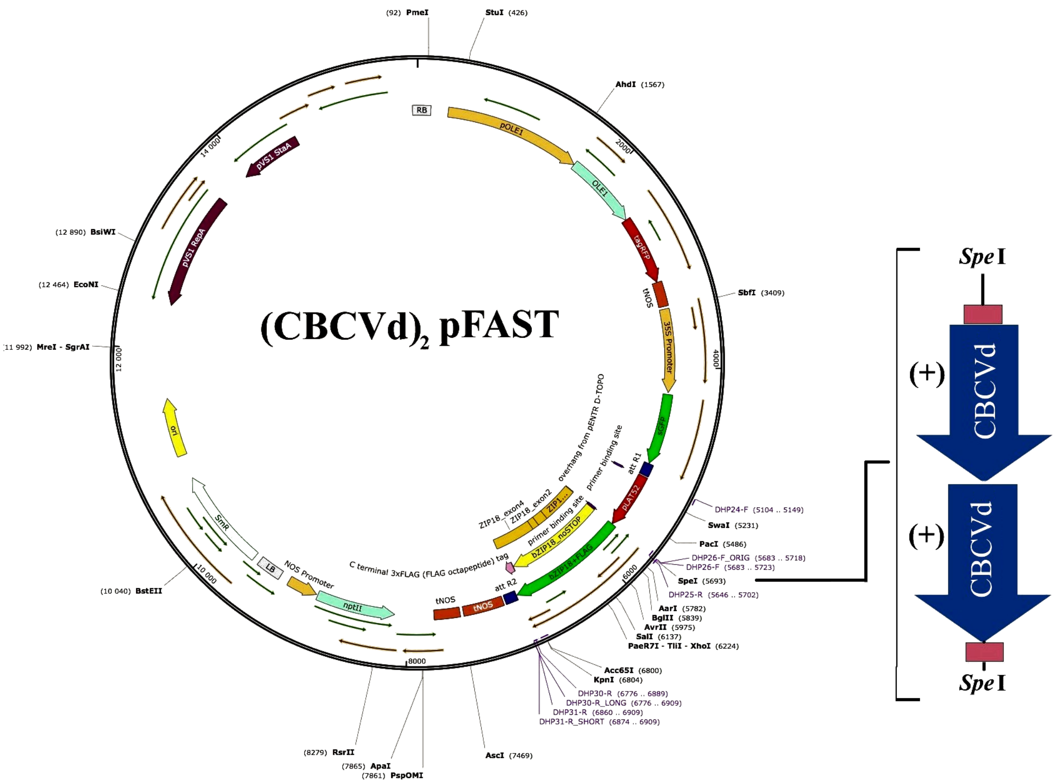

Figure S5. Plant vector cassettes used for transformation of *N. tabacum* with AFCVd (a) and CBCVd (b) infectious dimeric (++) cDNAs. Plant vector pFAST bearing late pollen-specific promoter pLAT52 described previously [1] was used for transformation. Infectious (++) dimers of AFCVd and CBCVd were integrated into unique *Kpn*I and *Spe*I restriction sites, respectively, downstream of pLAT52 promoter using specific adapters [1]. The vectors were transformed to *Agrobacterium* LBA4404 and used for tobacco transformation. The schemes are not to scale.

Table S1. Primers used for strand-specific RT-qPCR and quantification of mRNA levels by RT-qPCR.

No	Designation	Sequence 5′→3′	Purpose	Ref.
		*Nt*TFIIIA-9ZF, *Nt*TFIIIA-7ZF, *Nb*TFIIIA-7Z		
1	*Nt*TFIIIA_F2	GACATCTCTTGCAGCACCAAG	RT-qPCR	
2	*Nt*TFIIIA_R2	AACAAAGTGCCTCCATCGTC		
		AFCVd		
3	AFCVdRTPL	TCGTCGACGACGAGTCACCAGGTG	cDNA synthesis – reverse transcription	[5]
4	AFCVdRTMI	GTGACTCGTCGTCGACGAAGGGTC		[5]
5	AFCVd PCR_FOR	CCGGTCGTGGATACCTAGGA	RT-qPCR	[5]
6	AFCVd PCR_REV	ACGCGGCCTTCGGTGTG		[5]
		CBCVd		
7	CVdRTPL	AAGCCTGGGAGGAACAACCCAAGAG	cDNA synthesis – reverse transcription	[5]
8	CVdRTMI	GGATCCCCGGGGAAATCTCTTCAG		[5]
9	CVd PCR_FOR	TCACTGGCGTCCAGCACC	RT-qPCR	[5]
10	CVd PCR_REV	AGGAAGAAGCGACGATCGG		[5]
		*Nt*PNI		
11	*Nt*PNI-F	AACGGCGACTTATCGGCACTC	RT-qPCR	
12	*Nt*PNI-R	TGGATTGCACCAGCCACACAC		
		*Nt*DICER-like homologues		
13	pollenDcl-F	GAGTGCATGAAACATATGATACAG	RT-qPCR	[1]
14	pollenDcl-R	GAGAACTCTCAAGAAGCMTTGA		[1]
		*Nt*AGO5		
15	1AGO5F	CAGCCTTCATCATCACAACG	RT-qPCR	[1]
16	1AGO5R	CGTCCAACAGTTCCGTATCC		[1]
		*Nt*TUDOR S1-like nuclease (*Nt*TSN)		
17	TunucF	GTGGATGAGCCATTTGCATG	RT-qPCR	[1]
18	TunucR	GATGCCTCAGAAGCACCAGG		[1]
		Senescence regulating factor *Nt*NAC080		
19	*Nt*NAC_for	TAACCAAATAGTCCCAGTTCAGAGC	RT-qPCR	[6]
20	*Nt*NAC_rev	GTAACTCCAAAGGCAATCAAGACTC		[6]
		Senescence marker *Nt*CP1		
21	*Nt*CP1_for	CAGTGGCTAATCAACCTGTTTCGG	RT-qPCR	[6]
22	*Nt*CP1_rev	ACACCACTTGAATAGAACTGGAAATCG		[6]
		*Nt*ACTIN2		
23	*Nt*Actin_For	ACCTCTATGGCAACATTGTGCTCAG	RT-qPCR	[6]
24	*Nt*Actin_Rev	CTGGGAGCCAAAGCGGTGATT		[6]
		*Nt*ACTIN		
25	ACT-F1	TTCTGTTCCAACCATCAATGA	RT-qPCR	[1]
26	ACT-R1	GTACCACCACTGAGGACAATGT		[1]
		7SL RNA		
27	primer-α	TGTAACCCAAGTGGGGG	Reverse transcription and RT-qPCR	[7]
28	anti-β	GCACCGGCCCGTTATCC	RT-qPCR	[7]

References

1. Matoušek, J.; Steinbachová, L.; Drábková, L.; Kocábek, T.; Potěšil, D.; Mishra, A.; Honys, D.; Steger, G. Elimination of viroids from tobacco pollen involves a decrease in propagation rate and an increase of the degradation processes. *Int. J. Mol. Sci.* **2020**, *21*, 3029, [http://dx.doi.org/10.3390/ijms21083029].
2. Jones, P.; Binns, D.; Chang, H.Y.; Fraser, M.; Li, W.; McAnulla, C.; McWilliam, H.; Maslen, J.; Mitchell, A.; Nuka, G.; et al. InterProScan 5: genome-scale protein function classification. *Bioinformatics* **2014**, *30*, 1236–1240, [https://doi.org/10.1093/bioinformatics/btu031].
3. Matoušek, J.; Kozlová, P.; Orctová, L.; Schmitz, A.; Pešina, K.; Bannach, O.; Diermann, D.; Steger, G.; Riesner, D. Accumulation of viroid-specific small RNAs and increase of nucleolytic activities linked to viroid-caused pathogenesis. *Biol. Chem.* **2007**, *388*, 1–13, [https://doi.org/10.1515/BC.2007.001].
4. Boratyn, G.; Camacho, C.; Cooper, P.; Coulouris, G.; Fong, A.; Ma, N.; Madden, T.; Matten, W.; McGinnis, S.; Merezhuk, Y.; et al. BLAST: a more efficient report with usability improvements. *Nucleic Acids Res.* **2013**, *41*, W29–33, [https://doi.org/10.1093/nar/gkt282].
5. Matoušek, J.; Siglová, K.; Jakše, J.; Radišek, S.; Brass, J.; Tsushima, T.; Guček, T.; Duraisamy, G.; Sano, T.; Steger, G. Propagation and some physiological effects of *Citrus bark cracking viroid* and *Apple fruit crinkle viroid* in multiple infected hop (*Humulus lupulus* L.). *J. Plant Physiol.* **2017**, *213*, [https://doi.org/10.1016/j.jplph.2017.02.014].
6. Li, W.; Li, X.; Chao, J.; Zhang, Z.; Wang, W.; Guo, Y. NAC family transcription factors in tobacco and their potential role in regulating leaf senescence. *Front. Plant. Sci.* **2018**, *9*, 1900, [https://doi.org/10.3389/fpls.2018.01900].
7. Matoušek, J.; Junker, V.; Vrba, L.; Schubert, J.; Patzak, J.; Steger, G. Molecular characterization and genome organization of 7 SL RNA genes from hop (*Humulus lupulus* L.). *Gene* **1999**, *239*, 173–183, [https://doi.org/10.1016/S0378-1119(99)00352-2].
